# Clinical and biochemical effects of the E139K missense mutation in the *TIMP3* gene, associated with Sorsby fundus dystrophy

**Published:** 2009-06-15

**Authors:** Z. Saihan, Z. Li, J. Rice, N.A. Rana, S. Ramsden, P.G. Schlottmann, S.A. Jenkins, C. Blyth, G.C. Black, N. McKie, A.R. Webster

**Affiliations:** 1 Moorfields Eye Hospital, London, UK; 2 Institute of Ophthalmology, University College London, London, UK; 3 The National Genetics Reference Laboratories, Manchester, UK; 4 Royal Gwent Hospital, Newport, Wales; 5 Academic Unit of Medical Genetics and Regional Genetic Service, St. Mary's Hospital, Manchester, UK; 6 Wellcome Laboratory for Biogerontology, Newcastle upon Tyne, UK; 7 Academic Unit of Ophthalmology, Manchester Royal Eye Hospital, Manchester, UK

## Abstract

**Purpose:**

To determine the phenotypic and biochemical characteristics of the p.E139K missense variant in tissue inhibitor of metalloproteinase 3 (TIMP3) associated with Sorsby fundus dystrophy (SFD).

**Methods:**

The coding regions and adjacent intronic sequence of *TIMP3* were amplified by polymerase chain reaction and then analyzed by bidirectional sequencing. Allele-specific PCR was used to determine the minimum allele frequency of the mutant allele in ethnically matched controls. Clinical examination and imaging of affected individuals with color fundus photography, scanning laser ophthalmoscope (fundal autofluorescence), and optical coherence tomography was performed. A mutant construct of the TIMP3 protein was created and expressed in human retinal pigment epithelium (ARPE19) cells, which were then assayed for oligomerization and intrinsic matrix metalloproteinase (MMP) inhibitory activity.

**Results:**

Three affected individuals from a family of Welsh origin each harbored one allele of the *TIMP3* missense variant c.415 G>A, (p.E139K), which was not identified in 534 ethnically matched control chromosomes and thus presumed pathogenic. The mutant protein was shown to dimerize in culture cells and retain its MMP inhibitory activity. Retinal examination was variable between eyes of affected individuals and between family members. Drusen-like deposits were common to all three affected individuals and yellow subretinal deposits, exudative maculopathy, and geographic atrophy were also observed. Optical coherence tomography (OCT) images of affected individuals demonstrated hyperreflectivity of the RPE-photoreceptor-choroid complex.

**Conclusions:**

The TIMP3 p.E139K mutation is another cause of SFD. It is the second *TIMP3* sequence variant reported that does not affect the number of cysteine residues in the mutant protein yet dimerizes in vitro. The clinical presentation of this family is in keeping with previous clinical reports of this disorder.

## Introduction

Sorsby fundus dystrophy (SFD; OMIM 136900), first described by Sorsby in 1949 [1] is a fully penetrant autosomal dominant degenerative disease that leads to bilateral loss of central vision due to subretinal neovascularization as well as pigment epithelial atrophy at the macula. The course of vision loss is often rapid and further loss of peripheral vision and nyctalopia may also be a feature [1-4]

Age of onset ranges from the second to the eighth decade with the majority of cases presenting during the third to fifth decades of life [5]. Clinical findings include exudative or atrophic lesions of the macula, drusen-like deposits at the level of Bruch's membrane, angioid streaks, and plaque-like deposits of yellow subretinal material. Prolongation of dark-adaptation may also be present [6,7].

SFD is caused by mutations in the tissue inhibitor of metalloproteinase 3 (*TIMP3*) gene [8], which encodes a protein that is constitutively secreted by retinal pigment epithelium (RPE) and incorporated into Bruch’s membrane [9,10]. Bruch’s membrane is a multilayered extracellular matrix (ECM) separating the RPE from the vascular choriocapillaris. Histopathological studies in donor eyes of SFD patients have revealed that the abnormal subretinal deposits observed clinically do correspond to deposition of protein and lipid staining material within Bruch’s membrane [4], which in SFD donor eyes stains strongly for TIMP3 protein [10].

The *TIMP3* gene is a member of a family of four genes that encode endogenous inhibitors of matrix metalloproteinases (MMPs), a group of zinc-dependent endopeptidases. The balance between proteolytic MMPs and TIMPs is integral to ECM remodeling [11]. The *TIMP* family members can be separated structurally and functionally into N-terminal and C-terminal domains, each containing three intramolecular disulphide bonds, linking 12 conserved cysteine residues within the molecule [12,13]. The N-terminal domains of the TIMP family are more conserved in structure and function and are required for metalloproteinase inhibition and the induction of apoptosis [14,15], while the C-terminal domains are involved in ECM binding and impart more individual characteristics to the TIMP family members [16]. The C-terminal domain of TIMP3 is also the site of all reported mutations resulting in SFD to date [6,8,17-24].

Eleven distinct mutations have been reported to cause SFD, nine of which result in the creation [6,8,17-20], or loss of a cysteine residue by truncation of the protein [25], or skipping of exon 5 of the gene due to a splice site mutation [21]. The exact nature of the initiating pathology in SFD remains unclear. Experimental evidence suggests that in most cases, mutant TIMP3 constructs are associated to the ECM, retain their MMP inhibitory activity, and form high molecular weight protein–protein aggregates thought to result from additional intermolecular disulphide bonds between the unpaired cysteine residues created [16,26-30]. This has led others to propose that it is the increased deposition of TIMP-3 in Bruch’s membrane, rather than the dysregulation of metalloproteinase inhibition, that is likely to be the primary initiating event in SFD [31].

However, not all studies of TIMP3 mutants support these findings. One report, for example, found the p.S156C- TIMP3 mutant retained its MMP inhibitory activity and lacked dimerization [32].

Moreover, two SFD-linked mutations, p.H158R [22] and p.E139K [23,24], the focus of this study, failed to generate an unpaired cysteine residue in the predicted mutant protein. This may indicate a disease mechanism independent from disulphide-mediated oligomerization.

## Methods

### Patient selection and clinical evaluation

**Figure 1:**
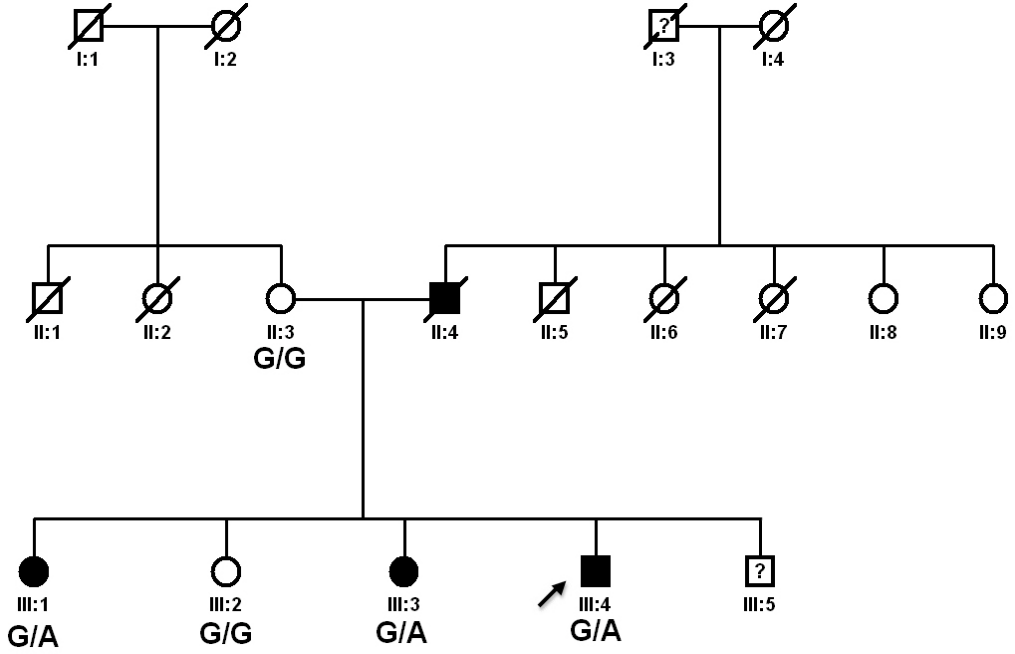
Pedigree of family showing genotypes for the c.415 G>A sequence variant in *TIMP3*. Affected individuals are shown in black. Genotypes for the c.415 G>A sequence variant (p.E139K) in *TIMP3* are shown underneath. Abbreviations: G/G represents homozygous wild type and G/A represents heterozygous for the mutant allele.

A three-generation British family of Welsh origin with an autosomal dominant history of retinal degeneration was referred to the Medical Retina clinic at Moorfields Eye Hospital (Figure 1). Three affected and two unaffected family members were available for study. The protocol of the study adhered to the provisions of the Declaration of Helsinki and was approved by the Moorfields Eye Hospital Ethics Committee. Genomic DNA was isolated from whole blood using an extraction kit (GE Healthcare Ltd, Buckinghamshire, UK). For the three affected individuals, full medical histories were taken and ophthalmic examination undertaken, including LogMAR visual acuities, slit-lamp biomicroscopy, color fundus photography, optical coherence tomography using 6-mm-length scans centered on the fovea (Stratus optical coherence tomography-OCT; Carl Zeiss Meditec, Dublin, CA), and fundus autofluorescence imaging, performed with a confocal scanning laser ophthalmoscope (cSLO, HRA, Heidelberg, Germany).

Images of fundus autofluorescence were recorded with a 40° field-of-view mode through a dilated pupil. The ametropic corrector was employed to correct for refractive error. An argon blue laser (488 nm) was used for excitation, and the emitted light of >495 nm was detected with a barrier filter. To amplify the autofluorescence signal, a flash mode was used. At least 24 single autofluorescence images of 512×512 pixels were acquired in series mode with a frequency of 12 images per second. The best 10 images were selected for automatic alignment and the creation of a single mean image for each eye.

### Mutation screening and sequence analyses

Exon 5 and flanking intronic sequences of TIMP3 were amplified from genomic DNA extracted from peripheral blood. Polymerase chain reactions (PCR; 25 µl) were performed using primer sequences described previously [8], containing the following: 200 ng genomic DNA, 12.5 µl 2X Mastermix (AB-0575, Abgene, Thermo Fisher Scientific, Leicestershire, UK), 3 µl of 2 mM forward and 3 µl of 2 mM reverse primer and 3.5 µl dH_2_O. Amplified PCR products were purified enzymatically using 1 µl ExoSAP-IT (GE Healthcare Ltd.) added to 5 µl of PCR product and 20 µl of dH_2_O. These samples were incubated at 37 °C for 15 min and heat inactivated at 80 °C for 15 min. Purified PCR product was then sequenced bidirectionally with 1 µl forward or reverse primer (2 µM stock), 1 µl BigDye (version 3.1) terminator cycle sequencing chemistry and 5 µl buffer (Applied Biosystems, Warrington, UK) as per the manufacturer’s protocol. The product was analyzed on an ABI 3100 Genetic Analyzer (Applied Biosystems, Applera, UK). The sequence was examined for alterations with Lasergene DNA Star software (DNA Star Inc., Madison, WI).

### Expression constructs of mutant TIMP-3

The reference coding DNA (cDNA) sequence of wild type *TIMP3* was derived from NCBI database (NM_000362) and the protein sequence from (NP_000353). As in previous reports of *TIMP3* sequence changes, the initial 69 nucleotides from the cDNA reference sequence (23 amino acids from the wild type protein sequence) were omitted from numbering; these nucleotides form a signal peptide for TIMP3 and do not form part of the functional molecule.

Wild-type pCDNA3 TIMP3 was used as the template and the forward primer 5′-TTG TGA CTT CCA AGA ACA AGT GTC TCT GGA CCG AC-3′, the reverse primer 5′-GTC GGT CCA GAG ACA CTT GTT CTT GGA AGT CAC AA-3′. The new construct, named pDNA3 E139K TIMP3, was confirmed by sequence analysis. The mutant construct was transfected in to ARPE-19 cells and incubated at 37 °C with 5% CO_2_ for 24 h. The medium was then changed into the selection medium containing Blasticidin S HCl at 5 μg/ml (Invitrogen, Carlsbad, CA). The cells were subcultured for 4 passages in 12 days in a T75 tissue culture flask. The ECM was harvested and protein sample was prepared. Nonreduced sample was separated on 15% polyacrylamide gels containing 0.09% SDS and transferred onto Polyvinylidene fluoride (PVDF) membrane. The membrane was incubated in blocking buffer (TBS, 0.1% Tween-20, 5% skimmed milk) for 12 h at 4 °C then probed with primary antibody (Sigma-Aldrich, St. Louis, MO; T7687) diluted 1:3,000 in blocking buffer for 16 h at 4 °C. The membrane was then probed with secondary goat anti-rabbit-HRP (P0448) antibody diluted 1:10,000 in blocking buffer at room temperature. The membrane was washed in TBS, 0.1% Tween-20 for 10 min following each antibody incubation. This process was performed three times. Protein was detected using ECL Advance Western Blotting Detection Kit (GE Healthcare UK Ltd, Little Chalfont, Buckinghamshire, UK) according to the manufacturer’s instructions.

### Determining allele frequency in a control population

The prevalence of the p.E139K allele in a control population was assessed using a validated competitive allele-specific polymerase chain reaction. The control population consisted of 188 unrelated UK blood donors from the European Collection of Cell Cultures (ECCAC, Salisbury, UK) and 79 unrelated volunteers from the Institute of Ophthalmology, London, UK.

### Assay of MMP inhibitory activity in the E139K mutant

Protease/substrate SDS–PAGE (reverse zymography) of mutant protein was performed to ascertain inhibitory activity of the TIMP3 containing fractions identified. Gelatin retained in SDS–PAGE gel was used as an in situ proteinase substrate. The proteolytic activity was reversibly inhibited by SDS during electrophoresis and renatured in subsequent washes by exchange of SDS with a nonionic detergent (Triton X-100, Sigma-Aldrich). Gel was then incubated with MMP-2 as well as MMP-9 (gelatinase) for 3–4 h. Partial degradation of gelatin was seen apart from in those regions containing fractions of TIMP3 with preserved MMP inhibitory function. The gels were then stained with Coomassie blue; the dark blue bands locating TIMP activity were visualized against the pale blue background of partially degraded gelatin [25].

## Results

### Identification of a disease-causing sequence variant in exon 5 of *TIMP3*

**Figure 2:**
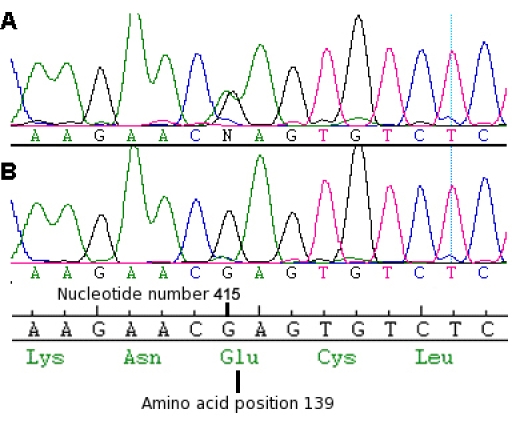
Electropherogram from affected family member III:4. Representative electropherogram from the sense strand of genomic DNA showing (**A)** heterozygous G>A missense change, (**B)** wild type sequence. The wild-type codon frame and amino acid number (139) is also shown.

The sequence variant c.415 G>A, resulting in a glutamate to lysine substitution p.E139K (Figure 2) was identified in all three symptomatic members of the family. This change was found to segregate with the disease (Figure 1).

### Allele frequency in control population

The sequence variant c.415 G>A, resulting in a glutamate to lysine substitution p.E139K (Figure 2) was identified in all three symptomatic members of the family. This change was found to segregate with the disease (Figure 1) and was not found in 534 ethnically matched control chromosomes.

### Clinical assessment

The index father (II:4) in the pedigree was deceased at the time of investigation. He reportedly suffered with progressive reduction in night vision starting in his third decade, with subsequent bilateral loss of central and peripheral vision by his fourth decade. The index mother (II:3) reported no ophthalmic history other than senile cataract and was not found to harbor the mutant allele. The clinical findings for affected individuals in this pedigree are summarized in Table 1.

**Table 1:** Clinical findings of affected individuals.

**Patient**	**Age onset of nyctalopia**	**Age of sudden vision loss OD**	**Age of sudden vision loss OS**	**Age at examination**	**Drusen-like deposits in posterior pole**	**LogMAR VA OD**	**LogMAR VA OS**	**Refraction OD**	**Refraction OS**
III:1	45	56	56	59	severe	3	0.6	(+1.00/+0.75x180)	(+3.00 ds)
III:3	45	52	52	53	moderate	0.48	1	(-1.5/+0.75x130)	(plano)
III:4	47	48	not applicable	51	occasional	0.96	-0.8	(+1.00/+0.25x120)	(-0.5 ds)

Table summarizing clinical findings from the three affected individuals harbouring the p.E139K change in *TIMP3*.

The three affected subjects carrying the p.E139K mutation all reported progressive reduction in night vision and slow dark adaptation during their fifth decade (mean age of onset of nyctalopia=45.7 years; standard error of the mean=2.4 years). In each case these symptoms predated the onset of sudden vision loss in the first eye from between 1 to 11 years.

**Figure 3:**
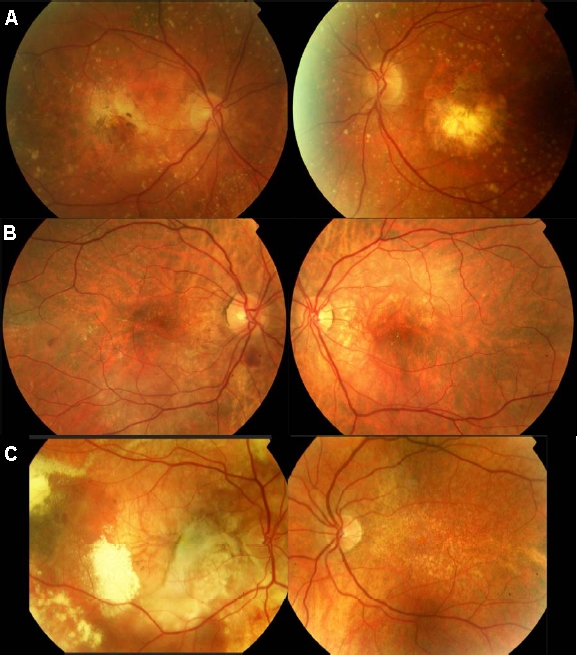
Color fundus photographs of the three affected individuals. All three affected individuals demonstrated the presence of yellow drusen-like deposits bilaterally throughout their fundi, but to varying amounts. A: Individual III:1 had bilateral geographic atrophy and widespread subretinal drusen-like deposits throughout the posterior pole extending beyond the vascular arcades. B: Individual III:3 had subretinal and intraretinal fluid at the macula in the right eye extending inferiorly toward the temporal arcades. In the right eye, two extrafoveal foci of subretinal hemorrhage were also noted, inferior to the optic disc and temporal to the macula. In the left eye (poorest visual acuity), subretinal fluid and a pigment epithelial detachment were noted superior to the macula. Although not visible on this image, areas of chorioretinal atrophy were also noted nasal to the disc. C: Individual III:4 had a large subretinal fibrovascular scar visible in the right eye. Foci of subretinal hemorrhage, exudates, and secondary retinal detachment are also visible. Areas of floccular yellowish subretinal deposit can be seen throughout the fundus in the right eye. In the left eye (without visual loss) only small yellowish subretinal deposits can be seen throughout the posterior pole.

No anterior segment abnormalities or visually significant lens opacity were noted in any of the three affected individuals, and no history of renal or systemic disease was found to segregate with the disorder in this pedigree. Yellow drusen-like deposits were present in all eyes of the three affected individuals to varying amounts but most obvious in III:1 (see Figure 3).

Hemorrhagic choroidal neovascularization (CNV) was the initial cause of sudden central vision loss in all three cases. At the time of examination the two older affected siblings had bilateral central vision loss (III:1 and III:3). The youngest (III:4) had unilateral vision loss, with his left eye showing no signs of CNV and maintaining good visual acuity.

At the time of last examination, retinal appearance varied greatly between family members, with individual III:1 displaying an atrophic maculopathy, individual III:2 displaying intraretinal fluid and pigment epithelial detachment. Individual III:4 had a striking fundal appearance in his affected eye, demonstrating widespread floccular yellow subretinal deposits, a large retinal pigment epithelial tear and evidence of extrafoveal neovascularization (Figure 3).

**Figure 4:**
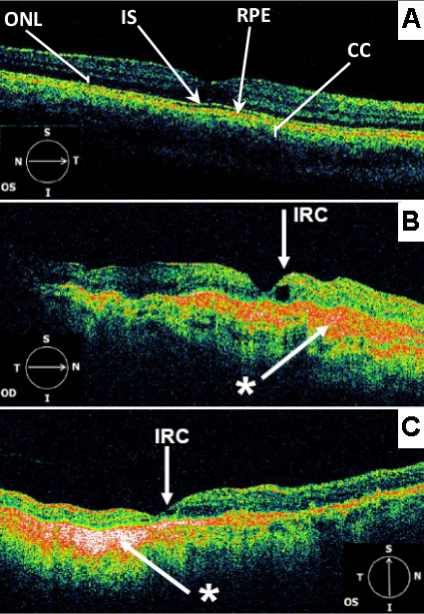
OCT3 scans (6 mm) centered on the fovea. (**A)** from a normal adult individual. Landmarks such as outer nuclear layer (ONL), inner and outer photoreceptor segment junction (IS), retinal pigment epithelium (RPE), and choriocapillaris (CC) are indicated. (**B)** from affected individual III:1 Right eye (OD) C: Individual III:1 Left eye (OS). Both **B** and **C** demonstrate hyperreflective signals (*) at the level of the RPE-choroid complex. The foveal pit appears steep in the right eye (OD). Parafoveal intraretinal cysts (IRC) are demonstrated bilaterally. There is also evidence of parafoveal retinal atrophy in the left eye (OS).

**Figure 5:**
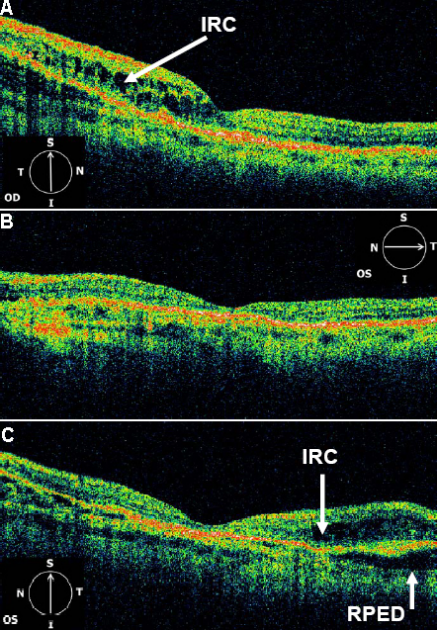
OCT3 scans (6 mm) centered on the fovea. From the right eye (**A**) and left eye (**B** and **C**) of affected individual III:3. The right eye (OD) demonstrates inframacular retinal thickening due to intraretinal cystic changes (IRC). The left eye (OS) demonstrates central retinal thinning and both retinal and pigment epithelial detachment (RPED) superior to the fovea. In the left eye there is hypereflectivity at the level of the choroid nasal to the fovea.

**Figure 6:**
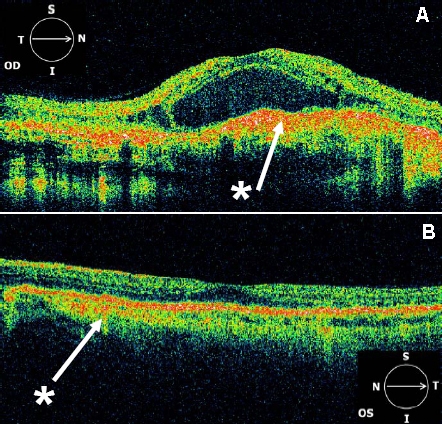
OCT3 scans (6 mm) centered on the fovea. From the right eye (**A**) and left eye (**B**) of affected individual III:4. Hyperreflectivity (*) at the level of the RPE-choroid complex is noted throughout the macula in the right eye (OD) and to a lesser extent in the left eye (OS). The right eye also demonstrates intraretinal cysts throughout the macula and a large central retinal detachment. The left eye demonstrates flattening of the foveal pit and loss of the outer nuclear layer temporal to the fovea.

OCT3 images from affected eyes displayed areas of RPE detachment and intraretinal cystic spaces (IRC), consistent with the choroidal neovascularization observed clinically (Figure 4, Figure 5, and Figure 6). An additional finding in both eyes of III:1 and III:4 was increased reflectivity at the level of the RPE-choroid complex. The left eye of III:4 did not have any evidence of CNV or vision loss, but the OCT scan showed evidence of retinal atrophy, in particular the loss of the outer nuclear layer temporal to the fovea (see Figure 6B).

**Figure 7:**
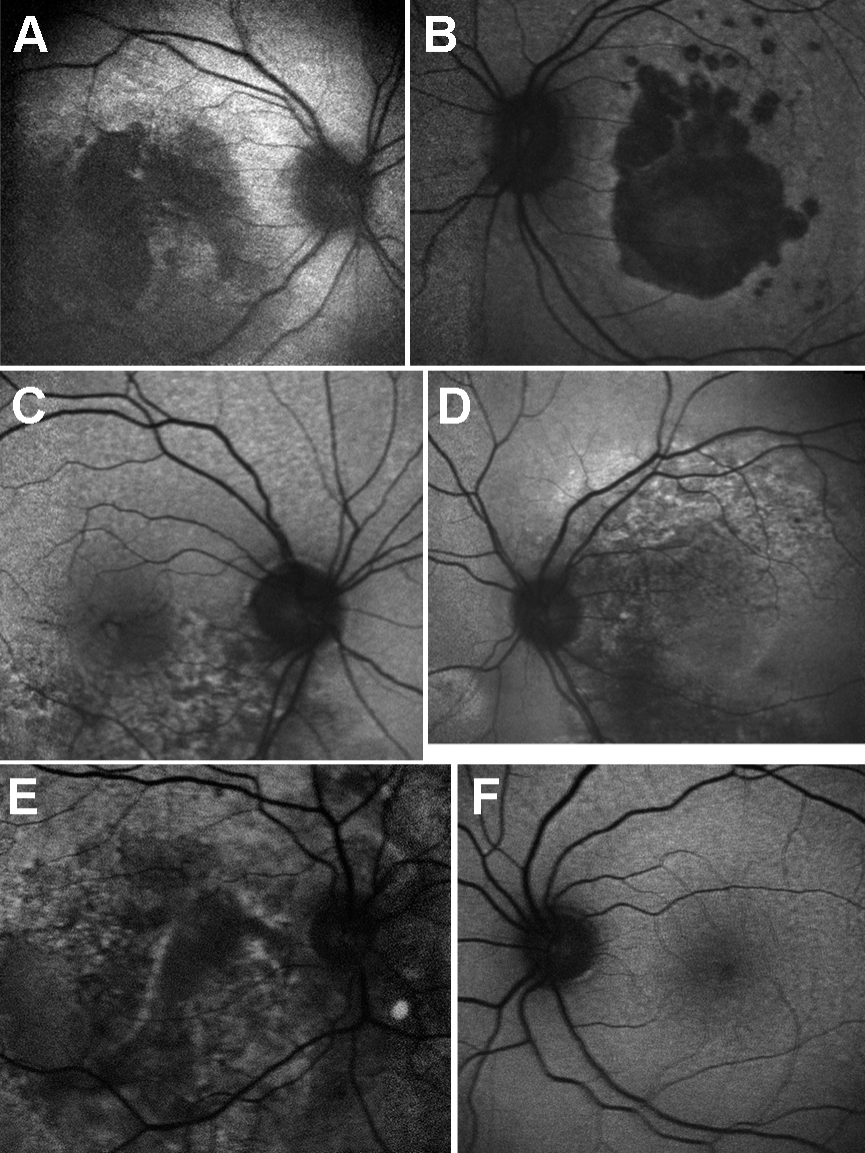
Fundus autofluorescence images of three affected individuals. **A** (right eye) and **B** (left eye) of individual III:1. Showing bilateral discrete areas of central confluent hypofluorescence associated with the areas of geographic atrophy noted clinically. The borders of this area are hyperfluorescent. **C** (right eye) **D** (left eye) of individual III:3. Bilateral areas of stippled hyperfluorescence and hypofluorescence extending toward the vascular arcades corresponding to the areas of subretinal fluid noted clinically. **E** (right eye) **F** (left eye) of individual III:4. In the right eye, a large stippled area of hyperfluorescence and hypofluorescence extending beyond the vascular arcades was noted. Of note, the areas of floccular yellow deposits seen on funduscopy were hypofluorescent on fundus autofluorescence imaging. Fundal autofluorescence in the left eye (without visual loss) was unremarkable.

Fundal autofluorescence imaging showed areas of irregular hyper and hypofluorescence in all eyes with vision loss. In the eye without vision loss (III:4), fundal autofluoresence did not reveal any significant abnormality (Figure 7).

### Expression construct and assay of MMP inhibitory activity of the p.E139K mutant

**Figure 8:**
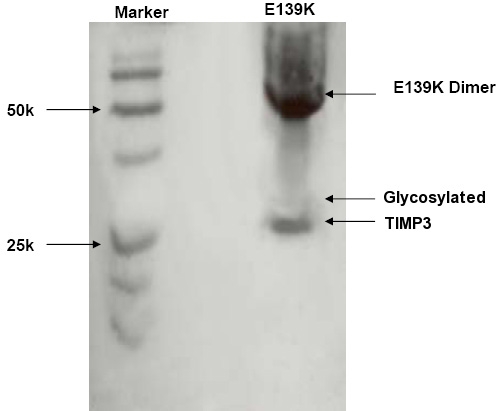
Western Blot of E139K TIMP-3 mutant. TIMP3 immunoblot (western blot) of the extracellular matrix sample from ARPE-19 cells transfected with E139K TIMP-3 expression plasmid, in nonreduced form demonstrating dimerization.

**Figure 9:**
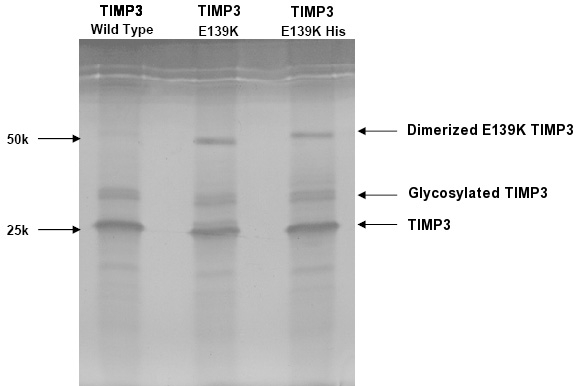
Reverse zymogram of extracellular matrix from the E139K TIMP3 mutant. The left lane demonstrates that the matrix metalloproteinase (MMP) inhibitory activity of the standard purified wild type TIMP3 is retained in both its glycosylated and nonglycosylated forms. Middle lane demonstrates that the TIMP3 E139K mutant also retains MMP inhibitory activity. Right lane represents the expression plasmid of E139K TIMP-3 with a polyhistidine tag, the addition of which resulted in increased molecular weight compared to the untagged construct in the middle lane.

Western blotting of the ECM extracts revealed a monomeric TIMP3 form in addition to a dimeric form at circa 48 kDa (Figure 8). The inhibitory activity of p.E139K TIMP3 mutant protein was tested by reverse zymography, using recombinant MMP-2 as an in-gel MMP activity. The dimerized TIMP3 p.E139K mutant could be seen as a high band with the size of 48 kDa in the gel with the presence of p.E139K TIMP3 monomer in the size of 24 kDa. Results of reverse zymography demonstrated that p.E139K TIMP3 mutant retained its MMP inhibitory activity and also could form a dimer within the ECM (Figure 9).

### Assay of MMP inhibitory activity in the p.E139K mutant

The inhibitory activity of p.E139K TIMP3 mutant protein was tested by reverse zymography, using recombinant MMP-2 as an in-gel MMP activity. The dimerized TIMP3 p.E139K mutant could be seen as a high band with the size of 48 kDa in the gel with the presence of p.E139K TIMP3 monomer in the size of 24 kDa. Results of reverse zymography demonstrated that p.E139K TIMP3 mutant retained its MMP inhibitory activity and also could form a dimer within the ECM (Figure 9).

## Discussion

The Welsh pedigree reported herein had clinical findings consistent with a diagnosis of SFD. Affected members segregated the c.415 G>A missense change in *TIMP3*, resulting in a glutamic acid to a lysine change at amino acid position 139 (p.E139K). This sequence variant was not found in 534 control chromosomes and thus was thus considered highly likely to cause the retinal disease in this family. This change has also been reported independently in a Swiss pedigree with SFD [24].

Ocular features in other pedigrees with SFD show interfamilial and intrafamilial variation, the reasons for this being unclear. The majority of SFD-causing mutations give rise to an unpaired cysteine residue and onset of disease between the third and fourth decade of life. There appears to be some relationship between mutation and age of vision loss, with some *TIMP3* mutations, such as p.S156C [17] and p.G166C [18,33], giving rise to vision loss as early as the second decade. Vision loss as late as the sixth to eighth decade has been reported in two Japanese pedigrees, segregating a splice site change [21]. Due to the lack of a proven theory explaining the biochemical mechanisms resulting in SFD, others have suggested that such phenotypic differences may relate to other genetic disease modifiers in affected pedigrees rather than the differences in mutations themselves [31].

The p.E139K reported here is the second reported *TIMP3* mutation causing SFD that fails to generate an unpaired cysteine residue, the other being p.H158R [22]. Of note, affected individuals from pedigrees harboring both these missense changes develop vision loss later in life (fifth to sixth decade) than in most other reported cases of SFD [22]. The reasons for this are unclear.

The propensity for mutant TIMP3 to form dimers has been explained by the presence of unpaired cysteine residues in these mutant molecules leading to aberrant intermolecular disulphide bonds [16,26-30]. Despite having an even number of cysteine residues, the p.E139K mutant protein expressed in ARPE-19 cells, forms dimers on western blotting and retains its MMP inhibitory activity. The change in charge at amino acid position 139, resulting from the positively charged lysine substituting the negatively charged glutamic acid may explain the dimerization of the p.E139K mutant observed. This change in charge may result in a disruption of disulphide bond formation at adjacent amino acid position 140. Further work would be required to test this hypothesis. Another hypothesis to explain the observed dimerization of the p.E139K mutant, could be the steric hindrance of the adjacent cysteine residue at amino acid position 140 by the substitution of lysine for a glutamate; however this is probably unlikely as both residues are of similar size.

An interesting finding in the OCT scans from the pedigree reported here was the abnormal hyperreflectivity noted at the RPE-choroid interface. A previous report of serial OCT scans in one individual with SFD showed a focal thickening of the RPE band at the location of subsequent CNV development [34]. Examination of SFD donor eyes have documented abnormal sub-RPE deposit and thickened Bruch’s membrane [4,35]. It is tempting to suggest that the sub-RPE hyperreflectivity demonstrated by this pedigree may represent the thickened Bruch’s membrane observed in donor eyes. However this hyperreflectivity may simply represent an artifact resulting from the increased light penetration due to overlying RPE atrophy or subretinal fibrosis from end stage CNV. Interestingly the OCT scan of the left eye (without central vision loss) from individual III:4 showed hyperreflectivity at the RPE-choroid interface but did not show any evidence of RPE disturbance on autofluorescence imaging (Figure 7E,F). This suggests that the hyperreflectivity may originate from below the RPE, perhaps from Bruch’s membrane.

Another interesting feature in the same OCT scan from the only eye without central vision loss was the retinal atrophy—specifically, loss of the outer nuclear layer in the perifoveal region. It is possible that atrophy of this rod-rich region of the retina may represent the early morphological changes related to the symptoms of nightblindness reported by this individual four years earlier.

In summary, we have described the detailed phenotype of an SFD family with a disease causing *TIMP3* mutation. The glutamic acid at position 139 in the TIMP3 molecule has previously shown to be mutated to a nonsense codon and is thus the first residue in the TIMP3 protein to cause SFD by two different classes of mutation. It is also the second sequence variant reported in this disorder that does not affect the number of cysteine residues in the predicted sequence of the TIMP3 protein, yet still exhibits dimerization in vitro. The mutant protein also retains its function as an MMP inhibitor.
